# Identifying causal models between genetically regulated methylation patterns and gene expression in healthy colon tissue

**DOI:** 10.1186/s13148-021-01148-9

**Published:** 2021-08-21

**Authors:** Anna Díez-Villanueva, Mireia Jordà, Robert Carreras-Torres, Henar Alonso, David Cordero, Elisabet Guinó, Xavier Sanjuan, Cristina Santos, Ramón Salazar, Rebeca Sanz-Pamplona, Victor Moreno

**Affiliations:** 1 Unit of Biomarkers and Susceptibility, Cancer Prevention and Control Program, Catalan Institute of Oncology (ICO), Av Gran Via 199-203, 08907 L’Hospitalet de Llobregat, Barcelona, Spain; 2 Colorectal Cancer Group, Bellvitge Biomedical Research Institute (IDIBELL), Hospitalet de Llobregat, Barcelona, Spain; 3 Biomedical Research Centre Network for Epidemiology and Public Health (CIBERESP), Madrid, Spain; 4 Program of Predictive and Personalized Medicine of Cancer (PMPPC), Germans Trias i Pujol Research Institute (IGTP), Badalona, Barcelona, Spain; 5 Department of Clinical Sciences, Faculty of Medicine, University of Barcelona, Barcelona, Spain; 6 Pathology Department, University Hospital Bellvitge (HUB), L’Hospitalet de Llobregat, Barcelona, Spain; 7 Medical Oncology Service, Catalan Institute of Oncology (ICO), Barcelona, Spain; 8 Biomedical Research Centre Network for Oncology (CIBERONC), Madrid, Spain

**Keywords:** DNA methylation, Genetics, Gene expression, mQTLs, eQTLs, eQTMs, Genetic and epigenetic control, Epigenetic regulation

## Abstract

**Background:**

DNA methylation is involved in the regulation of gene expression and phenotypic variation, but the inter-relationship between genetic variation, DNA methylation and gene expression remains poorly understood. Here we combine the analysis of genetic variants related to methylation markers (methylation quantitative trait loci: mQTLs) and gene expression (expression quantitative trait loci: eQTLs) with methylation markers related to gene expression (expression quantitative trait methylation: eQTMs), to provide novel insights into the genetic/epigenetic architecture of colocalizing molecular markers.

**Results:**

Normal mucosa from 100 patients with colon cancer and 50 healthy donors included in the Colonomics project have been analyzed. Linear models have been used to find mQTLs and eQTMs within 1 Mb of the target gene. From 32,446 eQTLs previously detected, we found a total of 6850 SNPs, 114 CpGs and 52 genes interrelated, generating 13,987 significant combinations of co-occurring associations (meQTLs) after Bonferromi correction. Non-redundant meQTLs were 54, enriched in genes involved in metabolism of glucose and xenobiotics and immune system. SNPs in meQTLs were enriched in regulatory elements (enhancers and promoters) compared to random SNPs within 1 Mb of genes. Three colorectal cancer GWAS SNPs were related to methylation changes, and four SNPs were related to chemerin levels. Bayesian networks have been used to identify putative causal relationships among associated SNPs, CpG and gene expression triads. We identified that most of these combinations showed the canonical pathway of methylation markers causes gene expression variation (60.1%) or non-causal relationship between methylation and gene expression (33.9%); however, in up to 6% of these combinations, gene expression was causing variation in methylation markers.

**Conclusions:**

In this study we provided a characterization of the regulation between genetic variants and inter-dependent methylation markers and gene expression in a set of 150 healthy colon tissue samples. This is an important finding for the understanding of molecular susceptibility on colon-related complex diseases.

**Supplementary Information:**

The online version contains supplementary material available at 10.1186/s13148-021-01148-9.

## Background

Over the past few years, multiple studies have shown that variation in germline genetics can modify DNA methylation levels, and subsequently, affect transcription and phenotypic variation [1–4]. These genetic variants are called methylation quantitative trait loci (mQTLs), in contrast to expression quantitative trait loci (eQTLs) that modify gene expression levels. Moreover, CpG sites and genes whose methylation and gene expression are correlated are known as expression quantitative trait methylation (eQTMs) [5].

To date, the extent at which DNA methylation is affected by genetic variation in colon tissue, as well as the extent of the genetically regulated gene expression that is mediated by methylation, remains unclear. Thus, solving the relations between genetic variants, methylation levels and gene expression levels may provide insight into the inter-individual variation of complex traits and diseases.

Although DNA methylation is often considered a repressive mark, its relationship with gene expression is complex. DNA methylation in promoters and enhancers is usually associated with transcriptional repression, while methylated CpGs located in the gene body are often associated with transcriptional activation and can also play a role in alternative splicing [6].

In this study, our objectives were to map common genetic variation affecting methylation levels (mQTLs) and methylation CpGs affecting gene expression levels (eQTMs) in healthy colon tissue and to identify causal relations between co-localizing mQTLs, eQTMs and eQTLs.

We analyzed 100 samples of normal colon tissue, adjacent to tumor, from patients with colon cancer and 50 samples of normal colon mucosae from healthy subjects. The analyses were centered in the group of samples that combined healthy mucosa donors and adjacent to tumor mucosa. We will call this group of samples Normal. Other exploratory analysis with Tumors were performed, and these were compared to their paired Adjacent normal samples only.

**Fig. 1:**
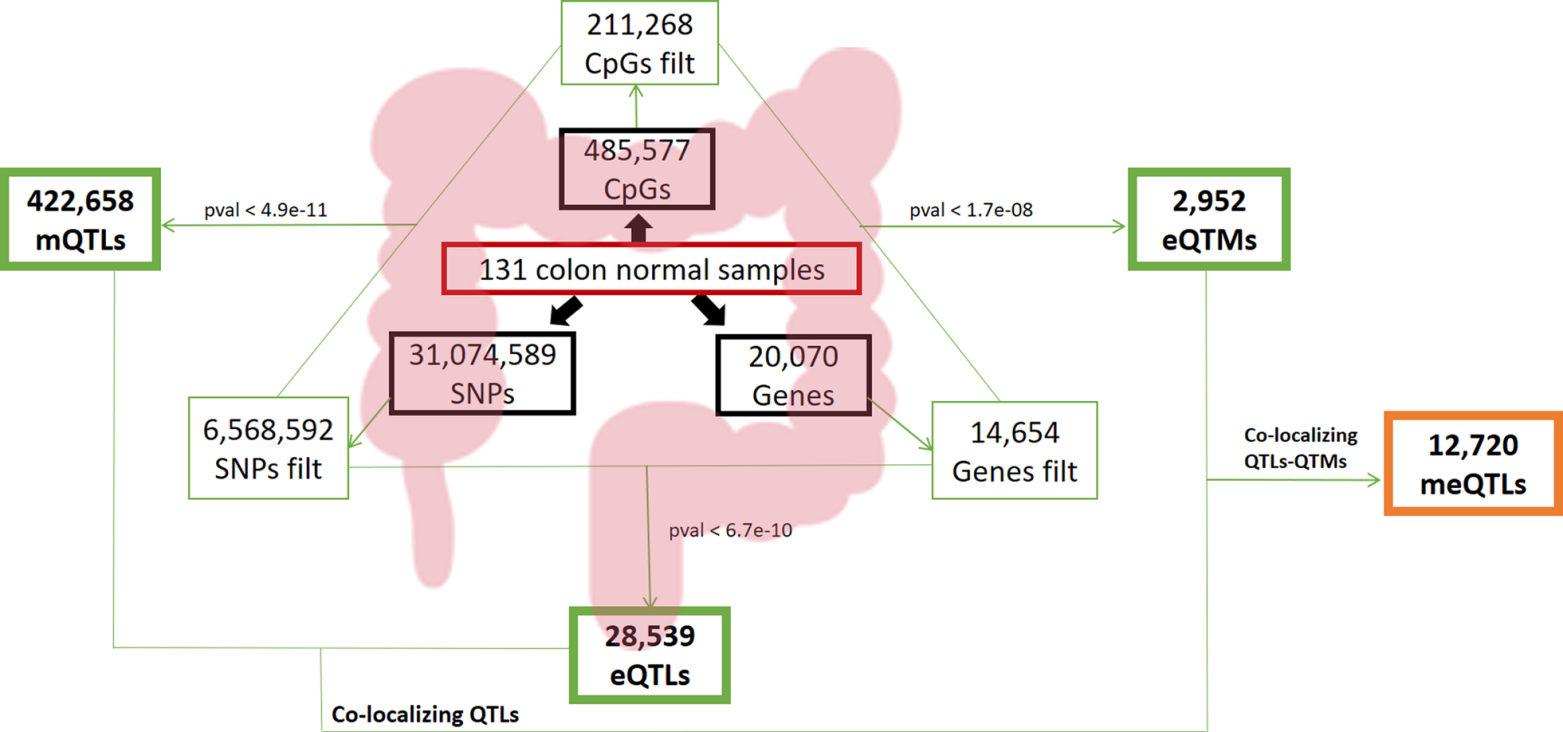
Scheme of the performed analysis. **SNPs filt**: Only SNPs in autosomal chromosomes and with a MAF between 0.05 and 0.95 have been considered. Duplicated SNPs and SNPs with more than 10% of missing values have been removed. **CpGs filt**: SNPs CpGs or CpGs with missing values have been removed. Only CpGs in autosomal chromosomes have been included. CpGs with a standard deviation greater than 0.05 have been filtered in. **Genes filt**: Only genes in autosomal chromosomes and with a standard deviation greater than 0.05 have been considered. All the analysis has been adjusted by sex, age and site and the maximum distance between elements was 1 Mb

**Fig. 2:**
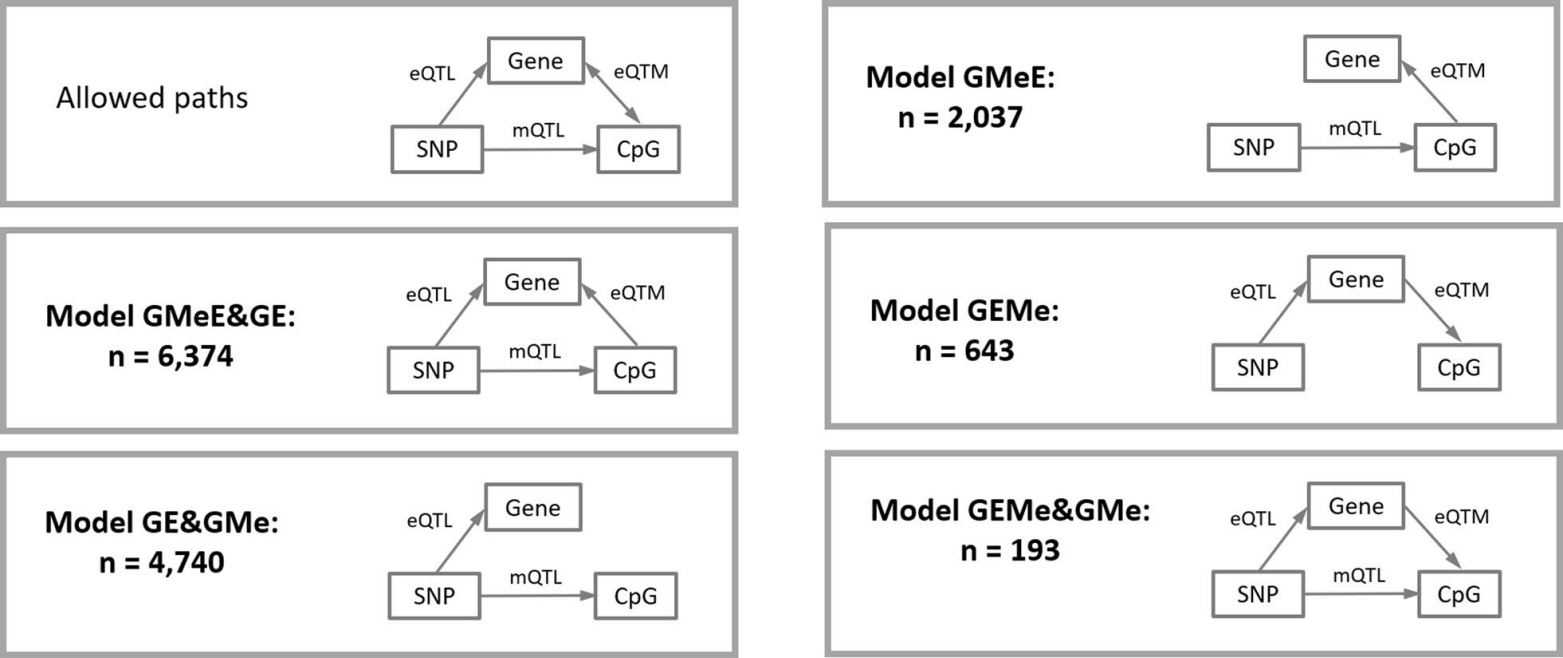
Causal relationship models of the 12,720 meQTLs in normal tissues

The samples of this study have been previously used to identify eQTLs [7] and also to profile DNA methylation which showed that DNA methylation in normal tissue of cancer patients was very similar to that of subjects without cancer [8]. In this study, we have assessed mQTLs and eQTMs and identified co-localizing triads of genetic variants, methylation sites and genes (meQTLs) (Fig. 1 and Additional File 1: Figure 1). Then we have classified these triads into different putative causal models using Bayesian network analysis (Fig. 2) and provided functional annotation of genetic variants associated with colon-related traits and diseases, such as colon cancer.

## Results

### Relationship between methylation and genetic variants (mQTLs) in colon tissue

We analyzed the possible association within 1 MB of 211,268 variable CpGs with 6,568,592 SNPs that passed quality control filters and detected 439,043 significant mQTLs (*p* < 4.9e−11). These involved 6713 CpGs (3.2%) and 246,758 SNPs (3.8%). The median distance between CpG and SNP was 47 Kb with a mean of 90 Kb and a standard deviation of 127 Kb. Since both nearby SNPs and CpGs are highly correlated, we identified 4524 and 8064 blocks of non-correlated cis-CpGs and cis-SNPs, respectively, and with these blocks we obtained a total of 8195 independent mQTLs (Table 1 and Additional File 2: Data 1).

**Table 1:** Number of significant associations identified

	Normal (*n* = 132)	Adjacent (*n* = 95)	Tumor (*n* = 95)
**mQTLs**	439,043	227,934	56,666
independent mQTLs	8195	4229	840
CpGs	6713	4167	850
CpGs blocks	4524	2845	645
SNPs	246,758	141,207	38,751
SNPs blocks	8064	4138	840
**eQTMs**	557	290	1732
independent eQTMs	165	78	490
Genes	158	78	487
CpGs	482	253	1563
CpGs blocks	155	75	466
**eQTLs**	32,446	17,274	8530
independent eQTLs	658	279	82
Genes	374	220	80
SNPs	31,482	17,070	8395
SNPs blocks	650	274	79
**meQTLs**	13,987	5517	1926
independent meQTLs	54	19	6
Genes	52	19	6
CpGs	114	45	16
CpGs blocks	51	19	6
SNPs	6850	2720	1231
SNPs blocks	54	19	6

Number of mQTLs, eQTLs, eQTMs and meQTLs. For each quantitative trait type, the number of genes, SNPs, CpGs, the number of independent quantitative traits (see methods) and the number of SNP and CpG blocks (elements in cis with *r*^2^ < 0.3, see methods). Normal corresponds to the group of samples that combines normal samples from healthy individuals (Healthy, *n* = 37) and normal mucosa adjacent to tumor (Adjacent, *n* = 95) from patients with cancer. Tumor corresponds to methylation analyzed in tumor tissue (*n* = 95).

**Fig. 3:**
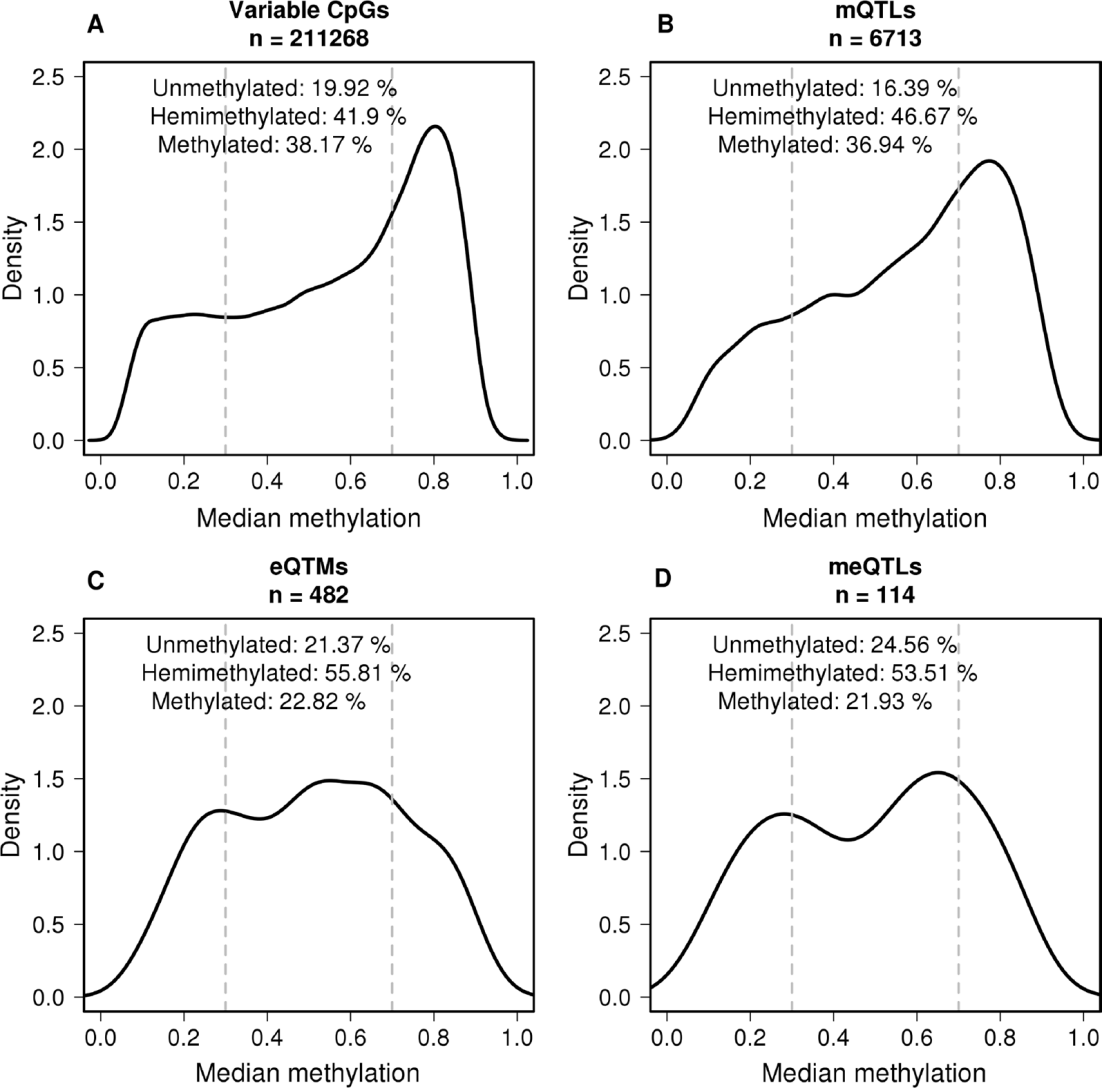
Methylation distribution in Normal tissues. Distribution of methylation median of **A** 211,268 variable CpGs, **B** 6713 CpGs in mQTLs, **C** 482 CpGs in eQTMs and **D** 114 CpGs in meQTLs

**Fig. 4:**
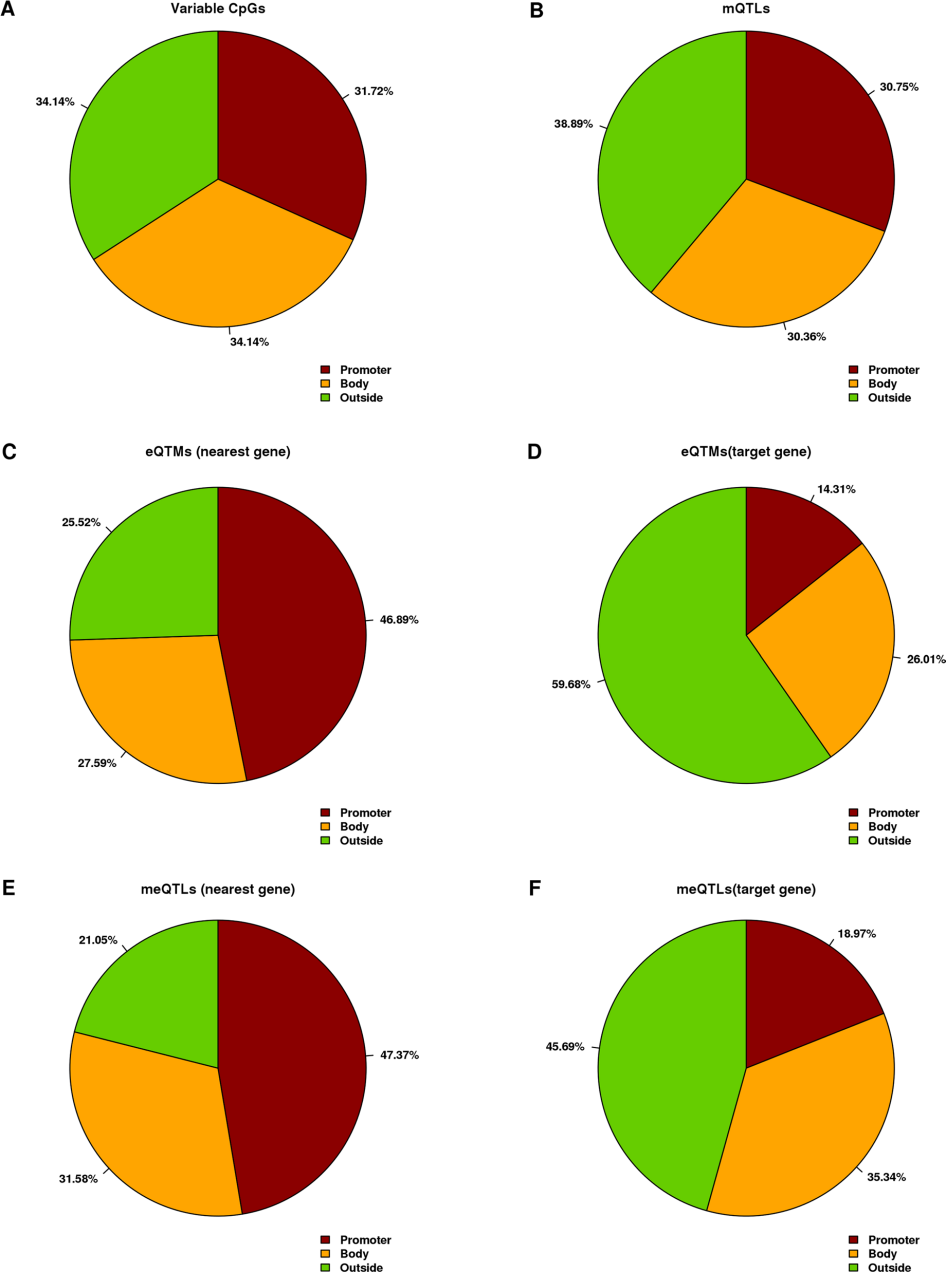
CpG distribution by gene region context. Proportion of CpGs by gene region context. **A** 211,268 variable CpGs associated with their nearest gene, **B** 6713 CpGs in mQTLs associated with their nearest gene, **C** 482 CpGs in eQTMs associated with their nearest gene, **D** 482 CpGs in eQTMs associated with the correlated gene, **E** 114 CpGs in meQTLs associated with their nearest gene and **F** 114 CpGs in meQTLs associated with the correlated gene

Additional File 3: Table 1 shows these global numbers by chromosome. We found an enrichment of non-correlated mQTLs in chromosome 6, possibly related to the human leukocyte antigen (HLA) hypervariable region. The distribution of mQTLs was similar to other variable CpGs regarding the distribution of median methylation levels (Fig. 3) and location in reference to genes (Fig. 4) and in reference to CpG island context (Additional File 4: Fig. 2).

### Relationship between methylation and gene expression (eQTMs) in colon tissue

Next, we found 557 eQTMs in normal colon tissues involving the expression of 158 genes (1.1% of the 14,654 genes) and 482 CpGs (0.2% of the variable CpGs). The median distance between the gene TSS and the CpGs was 168 Kb with a mean of 131 Kb and a standard deviation of 254 Kb. From these, we found 155 blocks of non-correlated cis- CpGs and 165 independent eQTMs (Table 1 and Additional File 5: Data 2). Additional File 3: Table 1 shows these global numbers by chromosome.

The CpGs involved in eQTMs showed a lower proportion of high methylation levels (Fig. 3A, C). The distribution of CpGs in eQTMs across the different regions of the gene context was very different if we looked at the nearest or at the target gene (Fig. 4C, D). When we looked at the nearest gene the proportion of promoter regions was very high (46.9%), while when we looked at the target gene, the proportion of promoter regions was very low (14.3%), most of the CpGs being outside the correlated gene (59.7%). The distribution regarding CpG island context, Additional File 4: Fig. 2C, is very similar to the distribution in variable CpGs and in mQTLs.

**Fig. 5:**
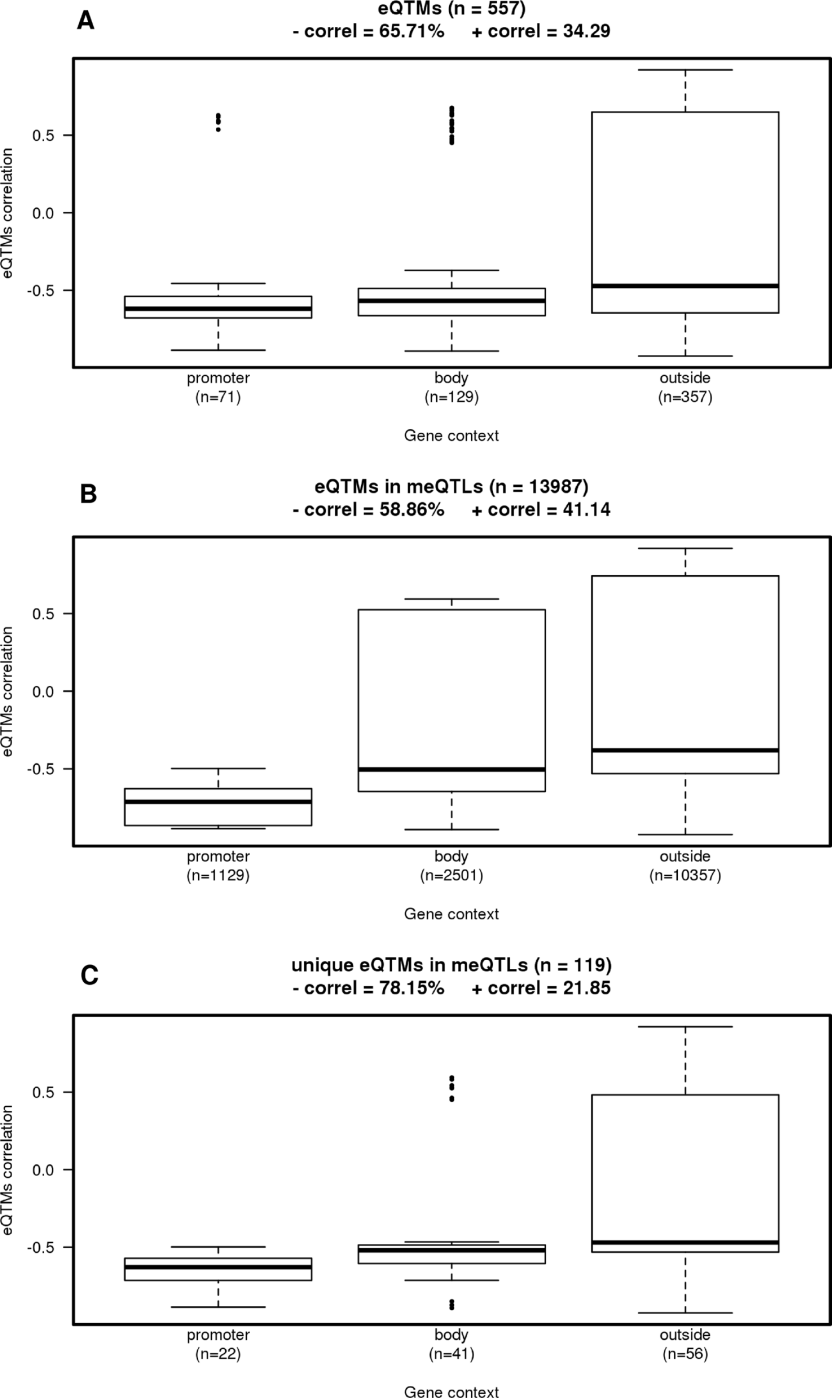
Boxplot of the correlation between gene and CpG by gene region context. **A** 557 eQTMs, **B** 13,987 eQTMs in meQTLs and **C** 119 unique eQTMs in meQTLs

There were slightly more eQTMs with a negative correlation between gene expression levels and CpG methylation levels (65.7%), and, as expected, the CpGs with a negative correlation were overrepresented in those CpGs that are inside the promoter of the associated gene (90.1%) and in the gene body (84.5%) (Fig. 5A).

### Co-occurring triads of associated genetic variants, methylation sites and gene expression levels

We had previously identified 32,446 eQTLs [7] (Table 1). We have now found that these eQTLs include 650 blocks of non-correlated cis-SNPs and 658 independent eQTLs (Additional File 5: Data 3).

We found 13,987 meQTLs, that is, triads of CpG, SNP and gene co-correlated in pairs of mQTLs, eQTMs and eQTLs. This involved 6850 unique SNPs, 114 unique CpGs and 52 unique genes; however, when linkage disequilibrium and correlation among neighbor CpGs were taken into account, there were only 54 and 51 independent blocks of cis-SNPs and cis-CpGs, respectively, with 54 independent meQTLs (Table 1). Additional File 3: Table 1 shows these global numbers by chromosome.

The distribution of the 114 CpGs involved in meQTLs showed less methylated CpGs compared with the rest of the groups (Fig. 3D). The proportion of CpGs in promoters (47.4%) was increased a little in comparison with the proportion in eQTMs, both, if we associated the CpG with the nearest gene (Fig. 4C, E) and if we associated the CpG with the significant correlated gene in eQTMs (Fig. 4D, F). The distribution of CpGs regarding CpG island context is very similar in all the groups (Additional File 4: Fig. 2).

Figure 5B shows the distribution of the correlation of 13,987 eQTMs in meQTLs by gene region context. 58.9% of the CpGs in meQTLs were negatively correlated with the expression of the gene. CpGs that had a positive correlation were mainly located outside the gene region. However, if we analyzed these same results but with the 119 unique eQTMs in meQTLs, 78.2% of the CpGs were negatively correlated with the gene and all the groups regarding gene region context showed a negative median correlation (Fig. 5C).

### Enrichment analyses of genes and SNPs in triads of genetic variants, methylation and gene expression

From the 6850 unique SNPs identified in meQTLs, only 718 (12%) mapped to regions associated with regulatory elements, specifically promoters and enhancers, in colonic mucosa, as indicated by predicted chromatin states and specific histone marks. Remarkably, compared to a subset of randomly sampled SNPs within 1 Mb of gene TSS, we found a significantly enrichment in SNPs mapping to these regulatory elements specially in those associated with promoters (Table 2 and Additional File 7: Fig. 3). As expected, these SNPs were also enriched in eQTLs.

**Table 2:** haploReg results

		eQTLs	mQTLs	meQTLs	Random SNPs	*p*-value eQTLs	*p*-value mQTLs	*p*-value meQTLs	*p*-adjusted eQTLs	*p*-adjusted mQTLs	*p*-adjusted meQTLs	Association	Name
	Total number	31,482	246,758	6850	150,000	–	–	–	–	–	–	–	–
Conservation	GERP	1049 (3.66%)	8112 (3.59%)	148 (2.35%)	6349 (4.65%)	5.04e−14	2.36e−55	8.57e−21	–	–	–	–	–
	Siphy	716 (2.5%)	5305 (2.35%)	116 (1.84%)	3619 (2.65%)	1.43e−01	1.29e−08	4.42e−05	–	–	–	–	–
Chromatin states	1_TssA	195 (0.68%)	1057 (0.47%)	32 (0.51%)	222 (0.16%)	1.03e−43	8.55e−57	1.19e−07	1.74e−42	1.45e−55	2.03e−06	Promoter-associated	Active transcription start site
	2_PromU	203 (0.71%)	1598 (0.71%)	62 (0.98%)	766 (0.56%)	3.78e−03	9.90e−08	8.23e−05	6.42e−02	1.68e−06	1.40e−03	Promoter-associated	Promoter upstream transcription start site
	3_PromD1	138 (0.48%)	841 (0.37%)	68 (1.08%)	375 (0.27%)	6.39e−08	7.11e−07	5.56e−19	1.09e−06	1.21e−05	9.46e−18	Promoter-associated	Promoter downstream transcription start site 1
	4_PromD2	129 (0.45%)	1195 (0.53%)	31 (0.49%)	300 (0.22%)	1.01e−10	5.71e−49	1.23e−04	1.72e−09	9.71e−48	2.10e−03	Promoter-associated	Promoter downstream transcription start site 2
	22_PromP	90 (0.31%)	924 (0.41%)	29 (0.46%)	306 (0.22%)	6.32e−03	1.71e−21	6.99e−04	1.08e−01	2.91e−20	1.19e−02	Promoter-associated	Poised promoter
	23_PromBiv	218 (0.76%)	2482 (1.1%)	47 (0.75%)	1227 (0.9%)	2.33e−02	5.59e−09	2.18e−01	3.96e−01	9.51e−08	1.00e + 00	Promoter-associated	Bivalent promoter
	10_TxEnh5	299 (1.04%)	2394 (1.06%)	83 (1.32%)	542 (0.4%)	1.08e−36	4.22e−114	8.44e−19	1.83e−35	7.17e−113	1.44e−17	Enhancer-associated	Transcribed 5′preferential and enhancer
	11_TxEnh3	417 (1.46%)	2332 (1.03%)	92 (1.46%)	474 (0.35%)	7.80e−92	5.23e−129	5.68e−27	1.33e−90	8.89e−128	9.66e−26	Enhancer-associated	Transcribed 3′preferential and enhancer
	12_TxEnhW	171 (0.6%)	840 (0.37%)	21 (0.33%)	259 (0.19%)	5.81e−28	2.28e−23	1.86e−02	9.87e−27	3.87e−22	3.15e−01	enhancer-associated	Transcribed and weak enhancer
	13_EnhA1	219 (0.76%)	1165 (0.52%)	111 (1.76%)	365 (0.27%)	4.68e−31	9.22e−31	1.30e−47	7.96e−30	1.57e−29	2.21e−46	Enhancer-associated	Active enhancer 1
	14_EnhA2	124 (0.43%)	1554 (0.69%)	54 (0.86%)	388 (0.28%)	8.46e−05	6.00e−64	2.32e−11	1.44e−03	1.02e−62	3.95e−10	Enhancer-associated	Active enhancer 2
	15_EnhAF	139 (0.49%)	1421 (0.63%)	30 (0.48%)	434 (0.32%)	3.16e−05	1.78e−39	4.04e−02	5.38e−04	3.02e−38	6.87e−01	enhancer-associated	Active enhancer flank
	16_EnhW1	155 (0.54%)	1136 (0.5%)	23 (0.37%)	271 (0.2%)	7.73e−21	9.90e−51	9.47e−03	1.31e−19	1.68e−49	1.61e−01	Enhancer-associated	Weak enhancer 1
	17_EnhW2	247 (0.86%)	1963 (0.87%)	26 (0.41%)	580 (0.43%)	8.48e−19	3.79e−58	1.00e + 00	1.44e−17	6.44e−57	1.00e + 00	Enhancer-associated	Weak enhancer 2
	18_EnhAc	110 (0.38%)	767 (0.34%)	32 (0.51%)	284 (0.21%)	2.27e−07	3.67e−13	1.43e−05	3.86e−06	6.24e−12	2.43e−04	Enhancer-associated	Primary H3K27ac–possible enhancer
	9_TxReg	129 (0.45%)	1274 (0.56%)	21 (0.33%)	807 (0.59%)	3.24e−03	2.97e−01	6.42e−03	5.50e−02	1.00e + 00	1.09e−01	Promoter/enhancer associated	Transcribed and regulatory
	19_DNase	110 (0.38%)	948 (0.42%)	38 (0.6%)	404 (0.3%)	1.68e−02	2.31e−09	1.06e−04	2.85e−01	3.93e−08	1.80e−03	Promoter/enhancer associated	Primary DNase
Histone marks	H3K4me3_Pro	3322 (11.6%)	27,570 (12.21%)	775 (12.3%)	7896 (5.79%)	7.20e−242	0.00e + 00	2.25e−79	2.88e−241	0.00e + 00	9.00e−79	Promoter-associated	Histone H3 lysine 4 trimethylation
	H3K9ac_Pro	4356 (15.21%)	30,036 (13.3%)	1207 (19.16%)	10,517 (7.71%)	7.45e−313	0.00e + 00	1.21e−175	2.98e−312	0.00e + 00	4.83e−175	Promoter-associated	Histone H3 lysine 9 acetylation
	H3K4me1_Enh	3019 (10.54%)	22,816 (10.1%)	794 (12.6%)	5877 (4.31%)	0.00e + 00	0.00e + 00	7.46e−146	0.00e + 00	0.00e + 00	2.98e−145	Enhancer-associated	Histone H3 lysine 4 monomethylation
	H3K27ac_Enh	5179 (18.08%)	43,529 (19.27%)	1097 (17.41%)	14,640 (10.73%)	1.83e−241	0.00e + 00	5.42e−54	7.31e−241	0.00e + 00	2.17e−53	Enhancer-associated	Histone H3 lysine 27 acetylation
eQTLs	eQTLs	20,199 (70.53%)	52,029 (23.04%)	5370 (85.22%)	6452 (4.73%)	0.00e + 00	0.00e + 00	0.00e + 00	–	–	–	–	–

Fisher exact test was used to compare, for each annotation in haploReg database, the proportion of SNPs in eQTLs, mQTLs and meQTLs with a random sample of cis-SNPs. Bonferroni adjusted *p*-values were calculated for chromatin states and histone marks separately.

Additionally, meQTLs were underrepresented among genetic conserved regions as indicated by GERP and SiPhy algorithms (*p*-value = 8.6e−21 and *p*-value = 4.4e−05, respectively) (Table 2).

The 52 genes found in meQTLs were mainly enriched in two groups of pathways, one related with metabolism of glucose and xenobiotics and other related with immune system through HLA genes (Additional File 8: Table 2).

A total of 64 SNPs involved in meQTLs (1% of 6850) were found associated with 48 traits from the GWAS (Table 3). It is interesting to note than all SNPs associated with circulating chemerin levels were statistically significant. From the 116 SNPs reported to be associated with colorectal cancer, three were identified as meQTLs: rs9271770, in cis with *HLA-DRB5* gene, within the 6p21.33 major histocompatibility region and associated with cg00119778; cg07984380 and cg15982117 CpGs; rs3087967 in the body of *c11orf52* and nearby rs3802842, an intronic variant of *COLCA1* and *COLCA2* in 11q23.1*,* were associated with the same CpG cg23091777.

**Table 3:** SNPs involved in meQTLs found in the GWAS catalog

	GWAS	Normal	Adjacent	Tumor
Traits	1722	48 (3%)	19 (1%)	15 (1%)
SNPs	48,881	64 (0.1%)	26 (0%)	15 (0%)
Age at menopause	84	1 (1%)	1 (1%)	0 (0%)
Alzheimer's disease (late onset)	55	1 (2%)	1 (2%)	1 (2%)
Asthma	206	2 (1%)	0 (0%)	1 (0%)
Asthma or allergic disease (pleiotropy)	36	1 (3%)	0 (0%)	0 (0%)
Blood metabolite levels	195	1 (1%)	1 (1%)	0 (0%)
Blood protein levels	2772	10 (0%)	7 (0%)	2 (0%)
Blood urea nitrogen levels	111	1 (1%)	0 (0%)	0 (0%)
Childhood ear infection	19	2 (11%)	2 (11%)	2 (11%)
Chronic lymphocytic leukemia	73	1 (1%)	0 (0%)	1 (1%)
Circulating chemerin levels	4	4 (100%)	0 (0%)	0 (0%)
Colorectal cancer	116	3 (3%)	1 (1%)	1 (1%)
Colorectal cancer or advanced adenoma	94	1 (1%)	0 (0%)	0 (0%)
Drug-induced liver injury (amoxicillin-clavulanate)	2	1 (50%)	1 (50%)	0 (0%)
Educational attainment (MTAG)	1320	1 (0%)	0 (0%)	0 (0%)
Eosinophil percentage of granulocytes	179	1 (1%)	1 (1%)	1 (1%)
Hair color	449	1 (0%)	0 (0%)	0 (0%)
Heart rate response to exercise	20	1 (5%)	1 (5%)	1 (5%)
Heel bone mineral density	2262	1 (0%)	0 (0%)	0 (0%)
High density lipoprotein cholesterol levels	306	2 (1%)	0 (0%)	0 (0%)
Highest math class taken (MTAG)	1084	1 (0%)	0 (0%)	0 (0%)
Intraocular pressure	512	2 (0%)	0 (0%)	0 (0%)
Liver enzyme levels	9	1 (11%)	0 (0%)	0 (0%)
Liver enzyme levels (gamma-glutamyl transferase)	26	2 (8%)	2 (8%)	1 (4%)
Lumiracoxib-related liver injury	1	1 (100%)	0 (0%)	0 (0%)
Mean platelet volume	323	1 (0%)	0 (0%)	0 (0%)
Medication use (adrenergics, inhalants)	55	1 (2%)	0 (0%)	0 (0%)
Menopause (age at onset)	63	1 (2%)	1 (2%)	0 (0%)
Metabolite levels	66	2 (3%)	0 (0%)	0 (0%)
Metabolite levels (small molecules and protein measures)	32	1 (3%)	0 (0%)	0 (0%)
Multiple sclerosis	158	1 (1%)	0 (0%)	0 (0%)
Multiple sclerosis (OCB status)	6	2 (33%)	1 (17%)	1 (17%)
Oligoclonal band status in multiple sclerosis	1	1 (100%)	1 (100%)	1 (100%)
Plasma homocysteine levels (post-methionine load test)	5	1 (20%)	1 (20%)	0 (0%)
Platelet count	323	1 (0%)	0 (0%)	0 (0%)
Plateletcrit	258	1 (0%)	0 (0%)	0 (0%)
Pulse pressure	747	1 (0%)	0 (0%)	0 (0%)
Red cell distribution width	821	1 (0%)	0 (0%)	0 (0%)
S-phenylmercapturic acid levels in smokers	1	1 (100%)	0 (0%)	0 (0%)
Serum metabolite levels	76	1 (1%)	1 (1%)	0 (0%)
Systemic lupus erythematosus	195	1 (1%)	1 (1%)	1 (1%)
Systolic blood pressure	1393	1 (0%)	0 (0%)	0 (0%)
Triglyceride levels in current drinkers	39	1 (3%)	0 (0%)	0 (0%)
Triglyceride levels x alcohol consumption (drinkers vs non-drinkers) interaction	55	1 (2%)	0 (0%)	0 (0%)
Triglyceride levels x alcohol consumption (regular vs non-regular drinkers) interaction	55	1 (2%)	0 (0%)	0 (0%)
Type 1 diabetes	87	1 (1%)	0 (0%)	1 (1%)
Type 2 diabetes	352	1 (0%)	1 (0%)	1 (0%)
Urinary 1,3-butadiene metabolite levels in smokers	2	2 (100%)	1 (50%)	1 (50%)
White blood cell count	854	2 (0%)	1 (0%)	0 (0%)

First column indicates the number of SNPs in each trait of the GWAS catalog, second to fourth columns indicate the SNPs in meQTLs found in each trait for Normal, Adjacent and Tumor groups, respectively. In parentheses, the percentage of SNPs in meQTLs that are in the list of SNPs associated to each trait.

### Putative causal relationships between methylation patterns, gene expression and their associated genetic variant

**Fig. 6:**
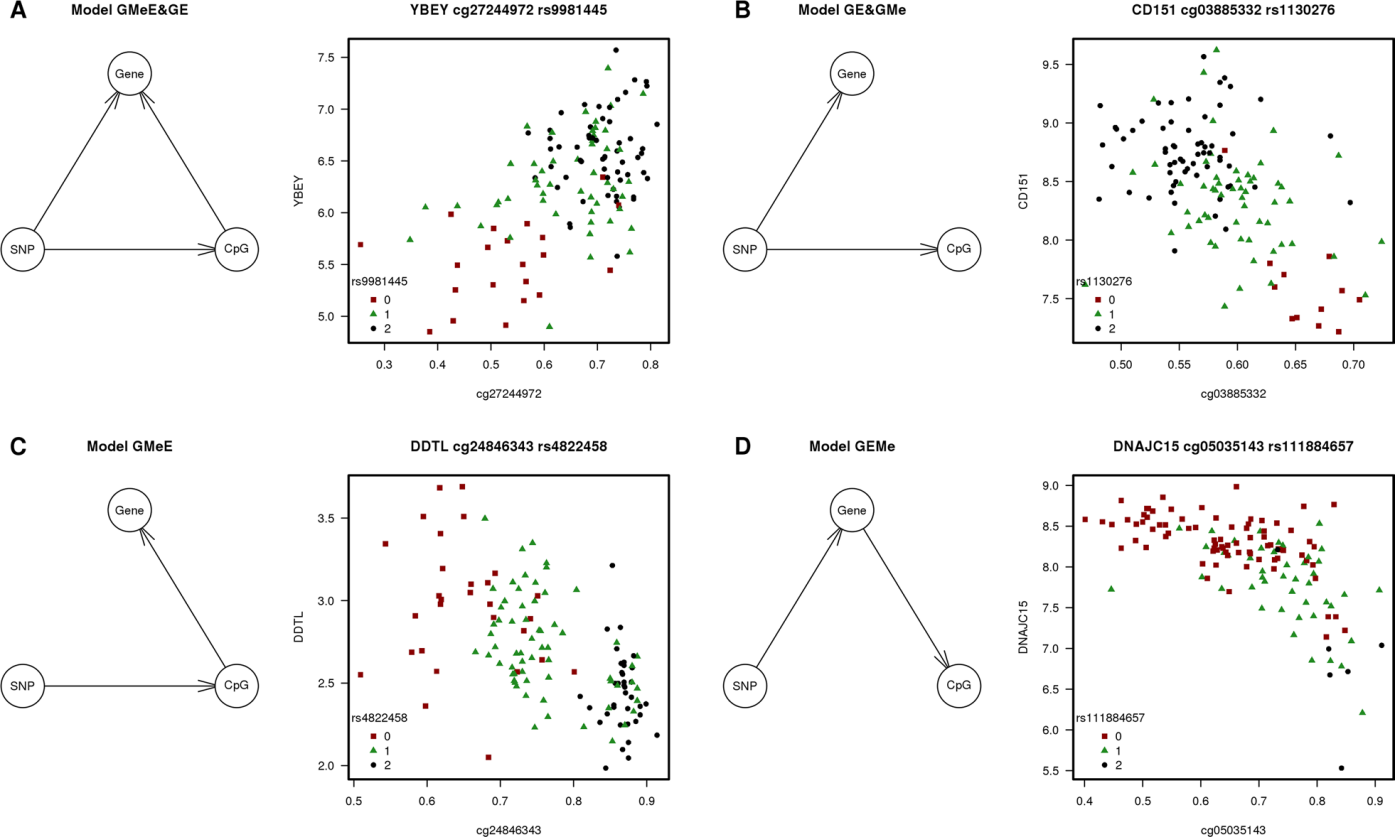
Examples of the models obtained from the Bayesian networks. First plot shows the diagram of the model and second plot shows the dotplot between methylation and gene expression taking genotypes as color legend

To study the putative causal relationship between genotypes, methylation and expression levels, we used Bayesian networks analysis to identify direct and mediated effects. We studied each of the 13,987 meQTLs triads and classified them into different models of causal relationships (Fig. 2). The most frequent model involved genetics (G) having a direct (putative causal) effect on gene expression (E) and at the same time, having an indirect effect on E through methylation levels (Me) (GMeE&GE model; 6374 meQTLs; 45.6%). As example, in Fig. 6A, the SNP rs9981445 had a direct effect on both cg27244972 CpG methylation and *YBEY* gene expression but, at the same time, the methylation of the CpG was also directly associated with gene expression of *YBEY*.

The following most frequent model consisted in a causal effect of G on both Me and E, with no relation between methylation and gene expression, indicating a passive role of DNA methylation (GE&GMe model; 4740 meQTLs; 33.9%). As example, in Fig. 6B, the SNP rs1130276 had a direct effect both on cg03885332 CpG and in *CD151* gene expression.

The third most frequent model described G influencing Me followed by an effect of Me on E levels, with no direct relation between G and E, indicating an active role for DNA methylation (GMeE model; 2037 meQTLs; 14.6%). An example of this causal model is shown in Fig. 6C where the SNP rs4822458 had a direct effect in cg24846343 CpG methylation and this CpG was affecting *DDTL* gene expression.

Finally, the last two causal models were scarcely present and involved E having a causal effect in Me. The most frequent among them was the model where G influenced E and E influenced Me (GEMe model; 643 meQTLs; 4.6%). The other model was the one where G influenced both E and Me and, at the same time, E, had a direct relationship with Me (GEMe&GMe model; 193 meQTLs; 1.4%). One example of GEMe model is shown in Fig. 6D where the SNP rs111884657 had a direct effect on gene *DNAJC15* and the gene expression was directly associated with the methylation of cg05035143 CpG.

Additional File 9: Table 3 shows how the 52 genes in meQTLs are distributed along the different models. Additional File 10: Fig. 4 shows the distribution of CpGs in meQTLs by gene region context along the different models. When we considered all CpGs in meQTLs (Additional File 10: Fig. 4A), we could see that GEMe&GMe model is the one with more proportion of CpGs in promoters followed by GMeE and GEMe models. On the other hand, if we analyzed unique CpGs in meQTLs (Additional File 10: Fig. 4B), the proportion of CpGs in promoters increased in all the models except in GEMe&GMe model. When we analyzed all the SNPs (Additional File 10: Fig. 4C) or unique SNPs (Additional File 10: Fig. 4D) in meQTLs we could see that, in both cases, most of the SNPs were outside the gene associated with the meQTL and there were very few SNPs in promoters. We also determined the enrichment of the SNPs in haploReg associated with promoter and enhancer-related chromatin states compared to a subset of randomly sampled SNPs for each model (Additional File 11: Table 4). Although we found few statistically significant enrichments, probably due to the low number of SNPs per chromatin state in each model, a proportion of the SNPs were located in predicted enhancers, especially in the GMeE&GE, GE&GMe models. The distribution of correlation of eQTMs in meQTLs is shown in Additional File 12: Fig. 5. If we considered all eQTMs, a positive median correlation was found in GMeE and GEMe models; however, if we considered unique eQTMs, all the models had a negative median correlation.

### Analysis of tumor tissue

To analyze whether tumor tissue has an altered regulation of gene expression mediated by methylation, we performed the mQTL, eQTLs and eQTMs analysis in 95 paired normal adjacent/tumors samples. In addition to the adjustment variables used for normal tissues, tumors were also adjusted by stromal content.

Table 1 shows the number of mQTLs, eQTLs, eQTMs and meQTLs in Normal, Adjacent and Tumor. We found that, with the exception of eQTMs, Normal group had a higher number of all the elements when compared with Adjacent or Tumor. This is possibly due to the larger statistical power of the combination of normal samples. Interestingly, Tumor had 3 and 6 times more eQTMs than Normal and Adjacent, respectively, but fewer other associations, indicating that gene expression and DNA methylation changes in tumors are highly correlated.

**Fig. 7:**
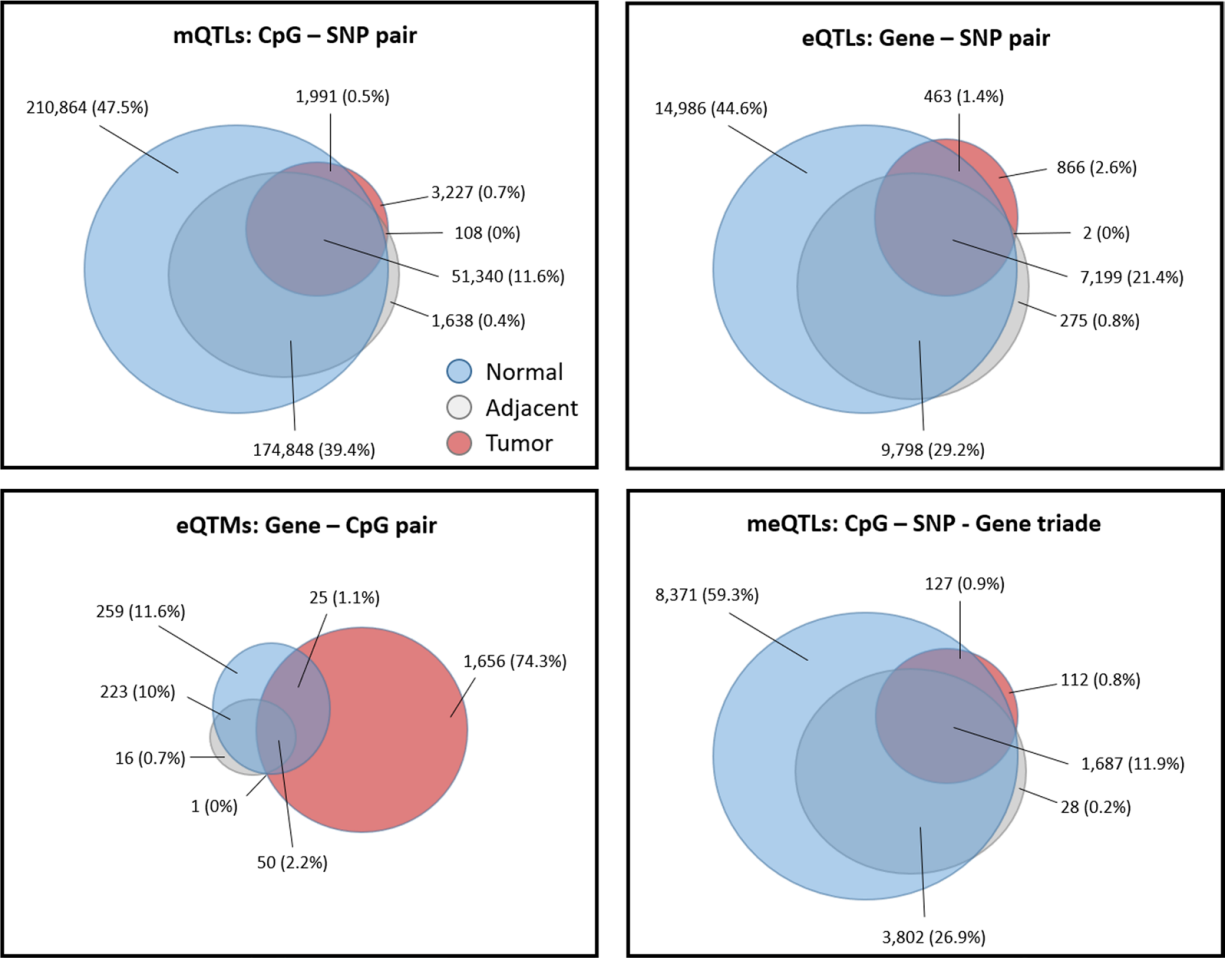
Common mQTLs, eQTLs, eQTMs and meQTLs between normal tissues and tumor samples. Venn diagrams of Normal tissue (healthy mucosae and adjacent normal tissue combined), Adjacent normal tissue alone and Tumor tissue

Figure 7 shows the Venn diagrams comparing the three groups of samples. All mQTLs, eQTLs and meQTLs had most common elements between the three groups of samples except the eQTMs of the Tumor group which had 74% of specific elements.

Additionally, we examined the overlap of the CpGs in mQTLs in Adjacent with those differentially methylated between normal adjacent tissue and tumor tissue found in [8]. We found that only 66 (1.6%) of the CpGs in mQTLs were differentially methylated in comparison to tumor tissue, as expected since our analysis is centered in normal tissues.

Finally, among CpGs and genes involved in eQTMs, only 12 (4.7%) of CpGs and 27 (34.6%) of genes were differentially methylated and differentially expressed, respectively, between tumor tissue and paired adjacent normal tissue in the differential analyses previously performed by our group [8].

We also performed the Bayesian network analysis to find putative causal relations between triads (Additional File 13: Table 5). The proportion of meQTLs in Adjacent normal tissue was very similar to the group Normal that combined adjacent normal plus healthy mucosae. Like Normal and Adjacent, most triads found in Tumors fitted with GMeE&GE model (46.6%) where a SNP affects expression directly and indirectly through methylation. GE&GMe and GMeE models lose some proportion of triads and, in contrast, models where expression explains methylation (GEMe&GMe and GEMe models) increased their proportion in Tumor.

## Discussion

Defining eQTLs, eQTMs, mQTLs and the co-occurring triads (meQTLs) in healthy colon tissue can help improve our knowledge of how genetic and epigenetic variation contribute to gene expression variation, which is important to better understand the etiology of colon diseases such as colon cancer and the inter-individual differences. As in other studies, we have focused on cis analysis as the majority of significant correlations have been found at less than 1 Mb of distance [7, 34, 35]. Little is known about the biological mechanisms that underlie meQTL effects. Here we report an approach to find meQTLs and explore using Bayesian networks whether the SNPs associated with methylation CpGs may have a causal role in gene expression changes.

Other studies that have explored the association between genetics, methylation and gene expression in blood cells also have found that mQTLs are the most abundant relationships and eQTMs the least abundant [11], suggesting that in colon, DNA methylation variation may be a less frequent mechanism than genetic variation regarding gene expression variation across individuals. Due to the limitation of the number of CpGs included in the 450 K array, further analyses using whole-genome or reduced-representation bisulfite sequencing should be performed to confirm this finding. The involvement of DNA methylation in gene expression is highly variable, by inhibiting the binding of transcription factors (TFs) [12–14], recruiting proteins that silence expression, such as methyl-binding proteins or histone deacetylases [15], regulating alternative intragenic promoters [16], or being influenced by TFs binding [17]. In this regard, Gutierrez-Arcelus et al. [11] also showed that the role of DNA methylation varied depending on the cell type. Whether DNA methylation is a consequence of gene regulation and plays a passive role, or whether it regulates gene expression and plays an active role, is still far from clear [15].

Similarly to other studies [36, 43] the majority of eQTMs, both associated and not associated with meQTLs, showed the canonical negative correlation between gene expression levels and CpG methylation levels, and, as expected, all the correlations of the CpGs within the promoter of the associated gene were negative. However, a notable proportion of CpGs located in the gene body or in intergenic regions showed a positive correlation, in line with many studies reporting that the function of DNA methylation varies with the genomic context [6].

Interestingly, although the CpGs in eQTMs and meQTLs were enriched in promoter regions of their closest gene, as a quantitative trait they were associated with another gene that was not the closest one, suggesting that these regions may act as enhancers regulating the associated gene. Another possible explanation would be that the methylation of the CpG indeed regulates the closest gene, but in a way that the association with gene expression is weak, but the product of this gene regulates the expression with other nearby gene for which we detect a stronger association in the eQTM [19, 20].

Most disease-associated SNPs are located in non-coding regions, and it has been shown for some of them that affect regulatory elements [21]. Accordingly, a functional analysis of the identified SNPs in meQTLs showed that they are enriched in promoter and enhancer chromatin states.

Interestingly, the analysis of the GWAS catalog revealed circulating chemerin levels function as highly significant. Recent works correlate concentration of chemerin with risk of colorectal cancer, thus suggesting these meQTLs could regulate intestinal homeostasis involved in carcinogenesis [22, 23]. Regarding gene functions, the most significant ones were those related with metabolism, specifically glutathione metabolism. Extensive literature links molecules in these pathways with colon carcinogenesis [49, 50]. Also, three meQTLs have been identified in colorectal cancer GWAS: rs9271770 related with *HLA-DQA1* [26], rs3087967 related with *C11orf53* [26] and rs3802842 [27] intronic to *COLCA1 and COLCA2* genes. The last two SNPs are located near each other in chromosome 11 and may not be completely independent because they share the same CpG cg23091777.

The number of mQTLs, eQTLs and meQTLs were lower when analyzed in tumors than their paired normal tissue, indicating a general deregulation of gene expression and methylation. The number of eQTMs, however, was larger in tumors and these eQTMs were specific for tumoral tissue, indicating that epigenetic but not genetic variation is an important factor driving gene expression variation in colon tumors in contrast to what occurs in normal colon tissue. This could be in part related to large chromosomal aberrations with copy number changes that might impact simultaneously methylation and gene expression.

Only a very small proportion of the CpGs involved in the mQTLs and eQTMs, 1.6% and 4.7%, respectively, are differentially methylated between Tumor and Adjacent normal, suggesting that the CpGs related to genetic and gene expression inter-individual variability in normal colon tissue are not directly involved in the colon tumorigenesis process. In contrast, the 34.6% of the genes involved in the eQTMs are differentially expressed between Tumor and Adjacent normal. However, the CpGs of the associated eQTMs are not differentially methylated between Tumor and Adjacent normal, suggesting that the mechanism underlying the altered expression of these genes in tumors is not DNA methylation, or at least it is not related to these CpGs.

In order to better understand the functional relationships between genetic variation, methylation and gene expression, we have used Bayesian networks. We assumed that the genetic component in these models (SNP) was driving the association with methylation and gene expression, and that the relationship between the latter two could be in either direction. Interestingly, we found that the most common causal relationship model was that in which the SNP affects expression both directly and indirectly through methylation (GMeE&GE model), followed by the model where the SNP affects methylation and expression independently of one another, thus DNA methylation having a passive role (GE&GMe model). This second model was also often found in fibroblasts and lymphoblastoid cell lines [11]. It is noteworthy that in both models there is a direct effect of the SNP on gene expression, reinforcing the predominant role of genetic variation on the inter-individual expression variability. The third most common causal relationship model was the mediation of DNA methylation (GMeE model), in which the SNP affects DNA methylation and DNA methylation in turn affects gene expression. This model was also observed in the analysis of T-cells [11]. The CpGs involved in these models show mainly a negative correlation with expression, which could be explained by the inhibition of TF binding or the recruitment of repressive proteins to regulatory elements by DNA methylation. Alternatively, DNA methylation can also create new binding sites for TF [28].

The models where the SNP affects methylation mediated by expression (GEMe and GEMe&GMe) are less frequent. The underlying mechanism may involve TFs whose binding to promoters would influence methylation so that when there is no binding DNA is accessible to be methylated [17]. Accordingly, these models are enriched in CpGs located in promoters. Interestingly, if we analyze the correlation between gene expression and methylation, considering the unique CpG-gene pairs in meQTLs, all models have a negative correlation.

Our analysis did not identify models in which DNA methylation and gene expression were unrelated to SNPs, showing other indirect associations. Though some of these relationships might exist in reality, probably we did not observe them because our analysis was restricted to QTLs.

### Study limitations

We have restricted our analysis to *cis*-associations, though there might be other significant eQTLs, eQTMs and mQTLs in *trans*. Though it has been reported in other tissues that long-distance relationships between SNPs, CpGs and genes exist [29], the biological interpretation would be difficult and probably most of those associations would be indirect effects.

Though there is high correlation in methylation among CpGs in islands, we opted to study associations at individual CpG level instead of at island level. This was because some studies have proven that a single differentially methylated CpG could affect gene expression [30]. To avoid inflating the number of findings due to redundancy, we identified blocks of contiguous correlated CpGs. Though some of these blocks are isolated CpGs in islands associated with gene expression, these findings, would require additional validation.

To ensure a robust analysis, we have been very strict both in the filters to include SNPs, CpGs or genes and in the *p*-value threshold to classify an eQTL, eQTM or mQTL as significant. If we compare our methodology with the one used in other papers, the list of significant eQTLs, eQTMs and mQTLs is smaller in our analysis and this may have made us discard some interesting results. The sample size of our study was limited, and we could not find other colon tissue datasets to validate the results or meta-analyze them.

Finally, it is well known that most DNA methylation variability is not genetically influenced, but related to environmental exposures such as smoking, diet or simply ageing. In fact, recent studies have identified signatures of CpGs whose global methylation status measures chronological age, known as the DNA methylation clock [31]. Thus, DNA methylation of eQTMs and mQTLs may vary with age affecting the interactions of DNA methylation with SNPs and gene expression.

## Conclusions

We have generated a comprehensive resource of DNA methylation variants in colon tissue which has allowed us to gain insight into the role of epigenetic variation in the interplay between genetic and gene expression variation. Results have shown a complex scenario in which the canonical relationship based on the influence of genetic variation on DNA methylation which in turn affects gene expression is not the unique, but DNA methylation can participate both in a passive and in an active manner. However, the factors determining the nature of this relationship are unknown, but they may be a combination of at least the cell/tissue type and the genomic location of the CpGs.

## Methods

### Aim

The aim of this study is to map genetic variation affecting methylation patterns (mQTLs) and methylation CpGs affecting gene expression (eQTMs) in healthy colon tissue. Using these two list of quantitative traits and the already published list of eQTLs [7], identify co-localizing triads of genetic variants, methylation sites and genes (meQTLs) and find causal relations between the elements in meQTLs using Bayesian networks.

### Colon tissue samples

Fresh tumor and paired adjacent normal mucosa samples of one hundred patients of colorectal cancer and fifty Healthy mucosa donors were included in the analysis. Sample recruiting and clinical characteristics of the samples can be found in [8, 32] but shortly, Healthy individuals had a mean age of 63 years while patients were 71 years old in mean. Half of the Healthy individuals were females, but only 28% among patients. All the colon cancer patients were diagnosed in stage II, received only radical surgery as treatment and tumors were microsatellite stable. Additional information about the study and patient samples can be found at [33].

### Genotyping data

Genotypes were obtained hybridizing genomic DNA extracted from colonic mucosa in Affymetrix Genome-Wide Human SNP 6.0 array (Affymetrix, Santa Clara, USA), which includes near 1 million single nucleotide polymorphism (SNP) markers. Genotype calling was performed with Corrected Robust Linear Model with Maximum Likelihood Classification (CRLMM) algorithm as implemented in R/Bioconductor package *crlmm* [34].

Whole genome imputation was performed using the IMPUTE2 software package [35] after haplotyping with SHAPEIT2 [36]. The 1000 genomes panel for CEU population, March 2012 version, was used as reference panel. We accounted for genotype imputation uncertainties by using an allelic dosage model. After imputation, SNPs were filtered out if the imputation quality info index was less than 0.4, the certainty index was less than 0.9 and the minor allele frequency (MAF) was less than 0.05. SNPs with more than 10% of missing data were also filtered out and only SNPs in autosomal chromosomes were considered. A total of 6,568,592 SNPs were included in the analysis. A total of 4 samples were excluded due to quality or sex concordance problems (3 Healthy and 1 Adjacent) so 146 samples (47 Healthy and 99 Adjacent) remained for the analysis.

### DNA methylation data

DNA methylation levels and differential methylation between samples (Tumor vs Adjacent and Adjacent vs Healthy) were previously assessed by our group [8]. In brief, DNA was extracted from colon mucosa specimens using the phenol–chloroform protocol. The extracted DNA was quantified using a Nano Drop ND 2000c spectrophotometer (NanoDrop Thermo scientific, Wilmington, DE) and stored at 4ºC. Bisulfite conversion of 600 ng of DNA was performed according to the manufacturer’s recommendations for the Illumina Infinium Assay (EZ DNA methylation kit. Zymo Research. Cat. No. D5004). The incubation profile was 16 cycles at 95ºC for 30 s, 50ºC for 60 min and a final holding step at 4ºC [37].

DNA methylation profiles were generated from the Illumina Human Methylation 450 K BeadChip assay. Technical details of this array are described elsewhere [38, 39]. This array interrogates methylation levels of 485,577 CpG sites. Array data were processed following a pipeline within the Bioconductor R environment. Library *minfi* was used for quality control and normalization [40]. Sample concordance was checked verifying the SNPs of the 450 K array with those of the Affymetrix Genome-Wide Human SNP 6.0 array (Affymetrix, Santa Clara, USA). Samples from 100 cancer patients and 39 Healthy donors were processed and after array quality control, six low-quality samples were excluded (2 Healthy and 4 patients), thus the final dataset analyzed contained data from 229 samples (37 Healthy, 96 Adjacent and 96 Tumor).

High-quality methylation probes were selected for analysis. Probes were excluded when signal detection *p*-value was > 0.01 for more than 5% of the samples. We discarded 41,082 probes that ambiguously mapped to multiple locations in the human genome with up to two mismatches [41]. We excluded 11,854 probes that contained SNPs within 10 bp. This resulted in a final set of 430,086 probes. We mapped the probe locations to the human genome sequence using UCSC genome browser (hg19) to retrieve an updated annotation of all genes. For the selected probes, a subset-quantile within array normalization (SWAN) was used to reduce systematic sources of bias known for this array [42].

At each CpG site, the methylation level was estimated as a β-value, which is the ratio of intensity signal obtained from the methylated allele over the sum of methylated and unmethylated alleles. M-values, the logit transformation of β-values, were used for the analysis, which increases the range of values in the extremes and reduces the dependency between mean and variance [43]. Probes outside autosomal chromosomes and with low variability were removed. Also, low variability probes were filtered. For that, a parametric-mixture cluster analysis on the standard deviation (sd) was used, and probes in the low variability clusters (sd < 0.05) were excluded (final *n* = 211,268, Additional file 14: Fig. 6). We used the sd and not the coefficient of variation (sd/mean) because that increased the apparent variability of very low methylated probes, which probably do not have a biological significance and would increase the likelihood of finding spurious associations [44].

Since principal component analyses revealed that adjacent normal mucosa samples clustered with samples from healthy individuals [8], adjacent normal and healthy mucosa samples were analyzed together in subsequent analyses (Normal).

### Gene expression data

Affymetrix Human Genome U219 Array Plate platform (Affymetrix, Santa Clara, CA, USA) was used to obtain gene expression data. Details are explained in [8], briefly, a block experimental design was performed to three 96-array plates to avoid batch effects. Robust Multiarray Average algorithm in *affy* package from R [45] was used to normalize data. After quality control 246 samples remain for the analysis (50 M, 98 N and 98 T). Genes with very low variability (standard deviation < 0.1 among all samples) and outside autosomal chromosomes were filtered out. A total of 14,654 genes remained in the analysis.

### Methylation quantitative trait loci

After quality control and considering only common samples between genotyping and methylation, a total of 132 samples (37 Healthy and 95 Adjacent) were used to identify mQTLs (Additional File 1: Fig. 1). To identify cis-mQTLs, each methylation CpG was correlated with SNPs within 1 Mb upstream and downstream methylation site (2 Mb overall). The genetic association was tested in a linear additive model (genotype dose vs methylation M-value) using the function modelLINEAR in R package *MatrixEQTL* [46] adjusting for age, colon tissue site (right/left) and gender. We used a *p*-value threshold of 4.9e−11 (0.05/211,268 CpGs × 4817 SNPs). The number of SNPs was calculated as the median of SNPs at a maximum distance of 1 Mb for each CpG (Fig. 1).

Independent mQTLs were calculated. First, blocks of correlated (*r*^2^ > 0.3) cis-CpGs were created and mQTLs were defined by these CpG blocks. After that, blocks of correlated (*r*^2^ > 0.3) cis-SNPs were created and mQTLs blocks were redefined based on these SNP blocks. For each mQTL block based on independent CpGs and SNPs, we choose the one with the minimum *p*-value as the representative mQTL of the block.

### Gene expression quantitative trait loci

In this analysis, we used 30,125 eQTLs found in this sample collection and reported in Moreno et al. [7]. Briefly, 144 samples (47 Healthy and 97 Adjacent) (Additional File 1: Fig. 1) were used and eQTLs were identified within a maximum distance of 1 Mb of the gene TSS (cis-eQTLs). A *p*-value threshold of 6.8e−10 (0.05/14,654 genes × 5000 SNPs) was applied. The number of SNPs was calculated as the median number of SNPs at a maximum distance of 1 Mb for each gene (Fig. 1).

Independent eQTLs were calculated. For each gene, blocks of correlated (*r*^2^ > 0.3) cis-SNPs were created and eQTLs were defined by these SNP blocks. For each eQTL block based on independent SNPs, we choose the one with the minimum *p*-value as the representative eQTL of the block.

### Gene expression quantitative trait methylation site

131 Normal samples (37 Healthy and 94 Adjacent) between gene expression and methylation were used to find the eQTMs performing the same analysis as for finding mQTLs (Additional File 1: Fig. 1). In the case of eQTMs, the association between methylation levels (M-value) and gene expression was tested adjusting by age, colon tissue site (right/left), tissue type (Healthy/Adjacent) and gender. A *p*-value threshold of 1.7e−08 (0.05/14,654 genes × 201 CpGs) was used. The number of CpGs was calculated as the median of CpGs at a maximum distance of 1 Mb for each gene (Fig. 1).

Independent eQTMs were calculated. For each gene, blocks of correlated (*r*^2^ > 0.3) cis-CpGs were created and eQTMs were defined by these CpG blocks. For each eQTM block based on independent CpGs, we choose the one with the minimum *p*-value as the representative eQTM of the block.

### Co-occurring triads of associated genetic variants, methylation sites and gene expression levels

To find co-regulation of methylation and expression levels by the same genetic variants (meQTLs), we searched for common SNPs among mQTLs and eQTLs, and then, we identified overlapping eQTMs (Fig. 1).

The number of independent meQTLs was calculated. For each gene, blocks of correlated (*r*^2^ > 0.3) cis-CpGs were created and meQTLs were defined by these CpG blocks. After that, blocks of correlated (*r*^2^ > 0.3) cis-SNPs were created and meQTLs blocks were redefined based on these SNP blocks. Finally, we count the number of meQTL blocks based on independent CpGs and SNPs for each gene.

### Functional annotation and pathway analysis

To annotate SNPs, the R package *haploR* [47] was used to query the HaploReg database [48]. HaploReg includes different types of annotation sources such as mammalian conserved regions (GERP and SiPhy algorithms), epigenetic marks (chromatin states (ChromHMM) corresponding to promoter or enhancer elements, specific promoter and enhancer histone marks) and eQTLs, for different cell and tissue types; in particular, we used data from colonic mucosa. We also submitted a random list of 150,000 cis-SNPs (within 1 Mb of gene TSS) that was used to calculate the expected distributions of each annotation. These were compared to the results of the meQTLs using a Fisher exact test. Bonferroni adjusted *p*-values were calculated for chromatin states and histone marks separately.

The R package *enrichR* [49, 50] was used to analyze for enrichment of the sets of genes tagged by meQTLs in different databases including KEGG [51], Reactome [52], GO [53] and MSigDB [54, 55].

### Enrichment in genome-wide association studies

We assessed whether the identified SNPs were associated with complex traits and diseases in European genome-wide association studies (GWAS) results from the GWAS catalog [56] using the *MRInstruments* package from R [57]. SNPs with a *p*-value greater than 5e−8 were filtered out from the catalog.

### Causal relations of meQTLs triads

Hill-climbing algorithm in *bnlearn* package from R [58] has been used to build a Bayesian network for each meQTLs triad. For that, M-values of methylation data, SNP dosage data and expression data were used. Blacklist parameter of the algorithm was used to avoid including the causal relation arcs where gene expression or CpG methylation explained the genetics of the SNP. The posterior probabilities for each potential causal model given by the Bayesian network analysis will allow us to identify the most probable causal relation in each meQTLs triad between the genetic variant, the methylation CpG and the levels of gene expression.

## Supplementary Information

**Additional file 1: Figure 1**. Number of samples in each data type and each quantitative trait analysis.

**Additional file 2: Data 1**. List of independent mQTLs.

**Additional file 3: Table 1**. For each chromosome, the number of elements (genes, CpGs and SNPs) analyzed, columns 1 to 3, and for each list of mQTLs, eQTMs, eQTLs and meQTLs, the number of elements, the number of unique elements and the number of non-correlated elements. Non correlated elements must be in cis and r^2^>0.3, see methods

**Additional file 4: Figure 2**. CpG distribution by CpG island context. Proportion of CpGs by CpG island context. A) 211,268 variable CpGs, B) 6,713 CpGs in mQTLs, C) 482 CpGs in eQTMs, D) 114 CpGs in meQTLs.

**Additional file 5: Data 2**. List of independent eQTMs.

**Additional file 6: Data 3**. List of independent eQTLs

**Additional file 7: Figure 3**. Proportion difference between random SNPs and SNPs in meQTLs in the Normal group for the different chromatin states. (*) indicates a significant enrichment or underrepresentation. X axis is: **1_TssA** - Active transcription start site; **2_PromU** - Promoter upstream transcription start site; **3_PromD1** - Promoter downstream transcription start site 1; **4_PromD2** - Promoter downstream transcription start site 2; **22_PromP** - Poised promoter; **23_PromBiv** - Bivalent promoter; **10_TxEnh5** - Transcribed 5′preferential and enhancer; **11_TxEnh3** - Transcribed 3′preferential and enhancer; **12_TxEnhW** - Transcribed and weak enhancer; **13_EnhA1** - Active enhancer 1; **14_EnhA2** - Active enhancer 2; **15_EnhAF** - Active enhancer flank; **16_EnhW1** - Weak enhancer 1; **17_EnhW2** - Weak enhancer 2; **18_EnhAc** - Primary H3K27ac–possible enhancer; **9_TxReg** - Transcribed and regulatory; **19_Dnase** - Primary DNase.

**Additional file 8: Table 2**. meQTLs functional analysis obtained with enrichR R package. Enrichment analysis of KEGG, GO and Reactome databases. For each term of the data base, the number of genes found in meQTLs/number of genes associated to the term (Overlap), the proportion test p-value, adjusted p-value and combined score and the genes in meQTLs that are included in the list of genes associated to the term.

**Additional file 9: Table 3**. Distribution of the 52 genes in meQTLs by each causal model.

**Additional file 10: Figure 4**: A) Proportion of CpGs, B) unique CpGs, C) SNPs and D) unique SNPs in meQTLs in the Normal group by gene region context along the different models.

**Additional file 11: Table 4**. haploReg results by model. Fisher exact test comparing, for chromatin states and histone marks annotation in haploReg database, the proportion of SNPs in each model with a sample of cis-SNPs. Bonferroni adjusted p-value was calculated for chromatin states and histone marks separately.

**Additional file 12: Figure 5**: Distribution of the correlation between CpGs and genes (eQTMs) in meQTLs (top) and unique eQTMs in meQTLs (bottom) for the Normal group along the different models.

**Additional file 13: Table 5**. Comparison of models produced with Bayesian networks between the three groups of samples (Normal, Adjacent and Tumor). For each group, the number of meQTLs, CpGs, SNPs and genes in each model.

**Additional file 14: Figure 6**. A) Mixture of normal distributions and clusters of CpGs according to the standard deviation (sd) of the beta-values. B) Distribution of CpGs according to the mean beta-value and standard deviation, with clusters colored. CpGs with sd < 0.05 were excluded from analysis.

## Data Availability

Both raw and normalized data of expression and methylation can be obtained from the Gene Expression Omnibus (GEO) database in project PRJNA188510, with accession number GSE44076 (gene expression) and GSE131013 (DNA methylation). The SNP data have been deposited in the European Genome-Phenome Archive under accession no. EGAD00010001253.

## Abbreviations

mQTL: Methylation quantitative trait loci
eQTL: Expression quantitative trait loci
eQTM: Expression quantitative trait methylation
meQTL: Co-occurring eQTM, eQTL and mQTL.
TSS: Transcription start site
TF: Transcription factor
Me: Methylation
E: Expression
G: Genetics
