# Supplementary figures and images for: Identifying causal models between genetically regulated methylation patterns and gene expression in healthy colon tissue
Source: Clin Epigenetics. 2021 Aug 21;13:162. doi: 10.1186/s13148-021-01148-9 (PMC8380335; doi:10.1186/s13148-021-01148-9)

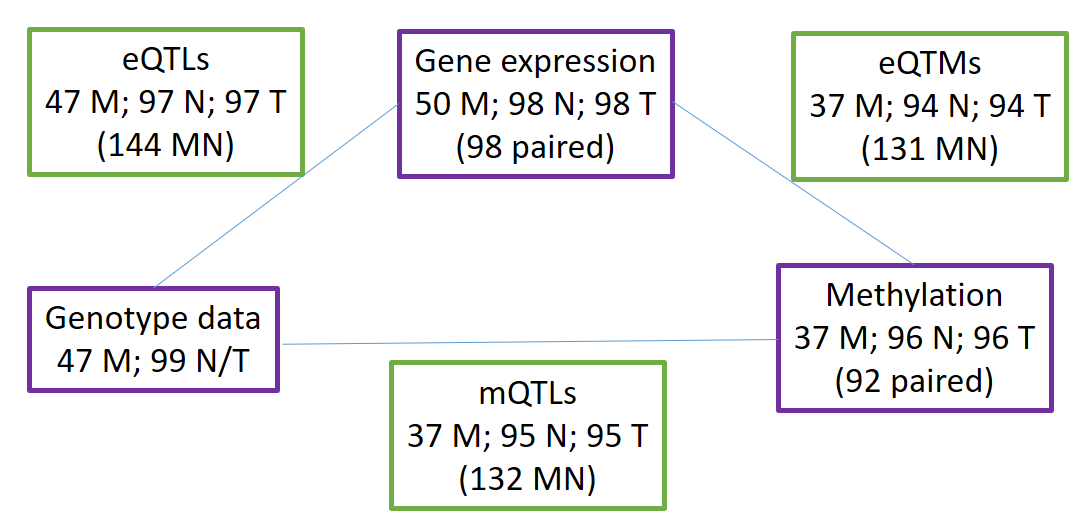

Supplement: Supplementary file 1 — Additional file 1: Figure 1. Number of samples in each data type and each quantitative trait analysis. [file 13148_2021_1148_MOESM1_ESM.png]

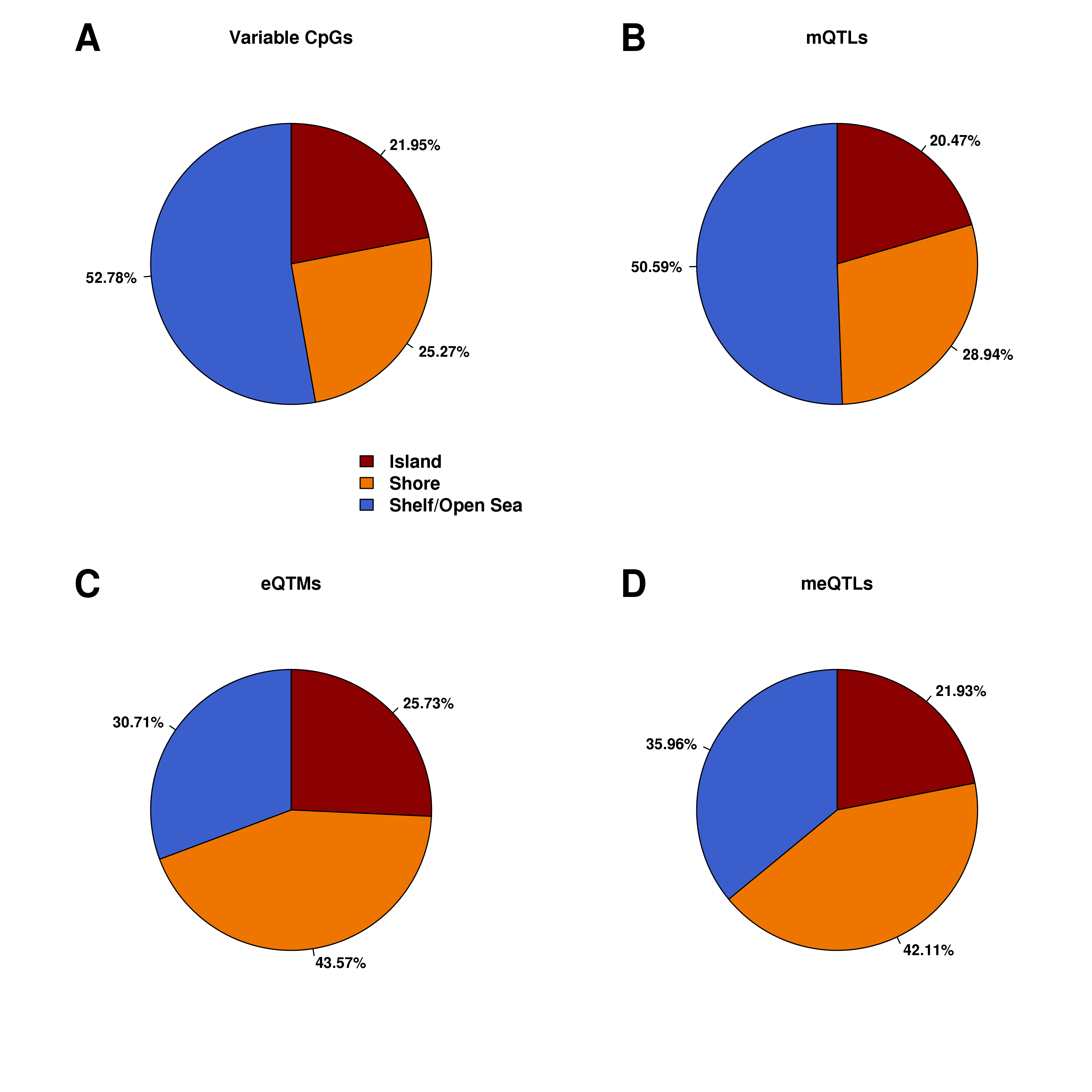

Supplement: Supplementary file 4 — Additional file 4: Figure 2. CpG distribution by CpG island context. Proportion of CpGs by CpG island context. A) 211,268 variable CpGs, B) 6,713 CpGs in mQTLs, C) 482 CpGs in eQTMs, D) 114 CpGs in meQTLs. [file 13148_2021_1148_MOESM4_ESM.png]

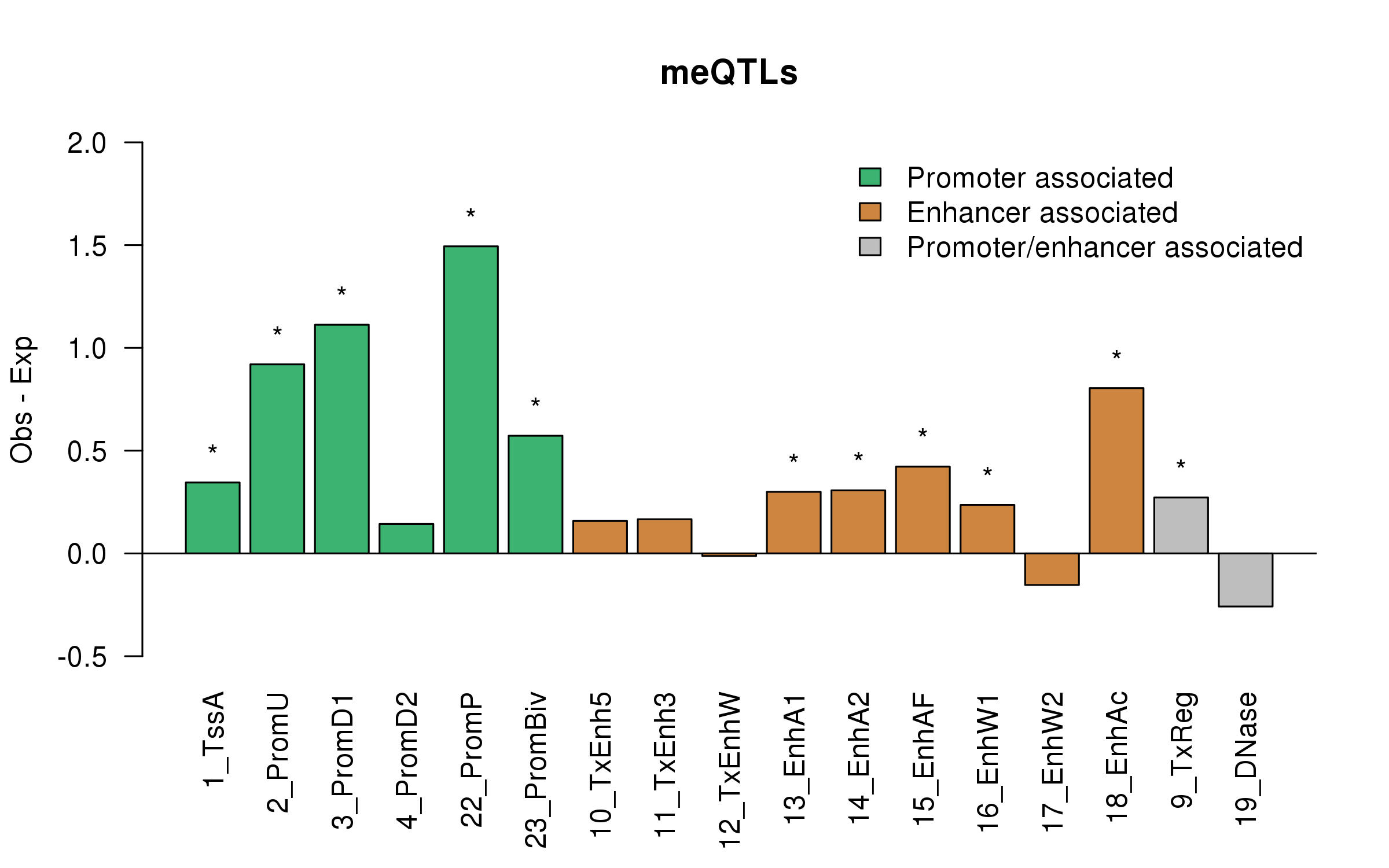

Supplement: Supplementary file 7 — Additional file 7: Figure 3. Proportion difference between random SNPs and SNPs in meQTLs in the Normal group for the different chromatin states. (*) indicates a significant enrichment or underrepresentation. X axis is: 1_TssA - Active transcription start site; 2_PromU - Promoter upstream transcription start site; 3_PromD1 - Promoter downstream transcription start site 1; 4_PromD2 - Promoter downstream transcription start site 2; 22_PromP - Poised promoter; 23_PromBiv - Bivalent promoter; 10_TxEnh5 - Transcribed 5′preferential and enhancer; 11_TxEnh3 - Transcribed 3′preferential and enhancer; 12_TxEnhW - Transcribed and weak enhancer; 13_EnhA1 - Active enhancer 1; 14_EnhA2 - Active enhancer 2; 15_EnhAF - Active enhancer flank; 16_EnhW1 - Weak enhancer 1; 17_EnhW2 - Weak enhancer 2; 18_EnhAc - Primary H3K27ac–possible enhancer; 9_TxReg - Transcribed and regulatory; 19_Dnase - Primary DNase. [file 13148_2021_1148_MOESM7_ESM.png]

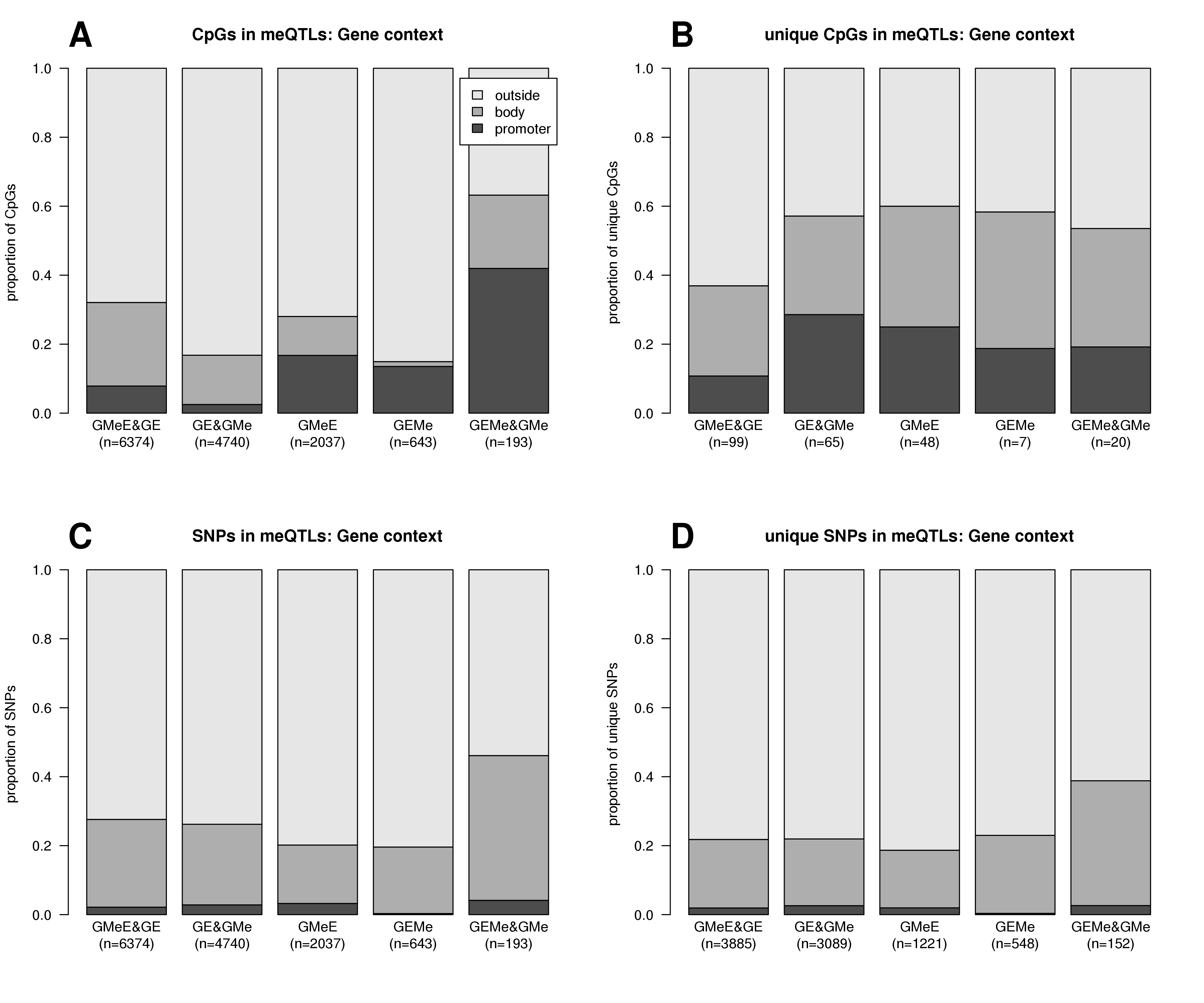

Supplement: Supplementary file 10 — Additional file 10: Figure 4: A) Proportion of CpGs, B) unique CpGs, C) SNPs and D) unique SNPs in meQTLs in the Normal group by gene region context along the different models. [file 13148_2021_1148_MOESM10_ESM.png]

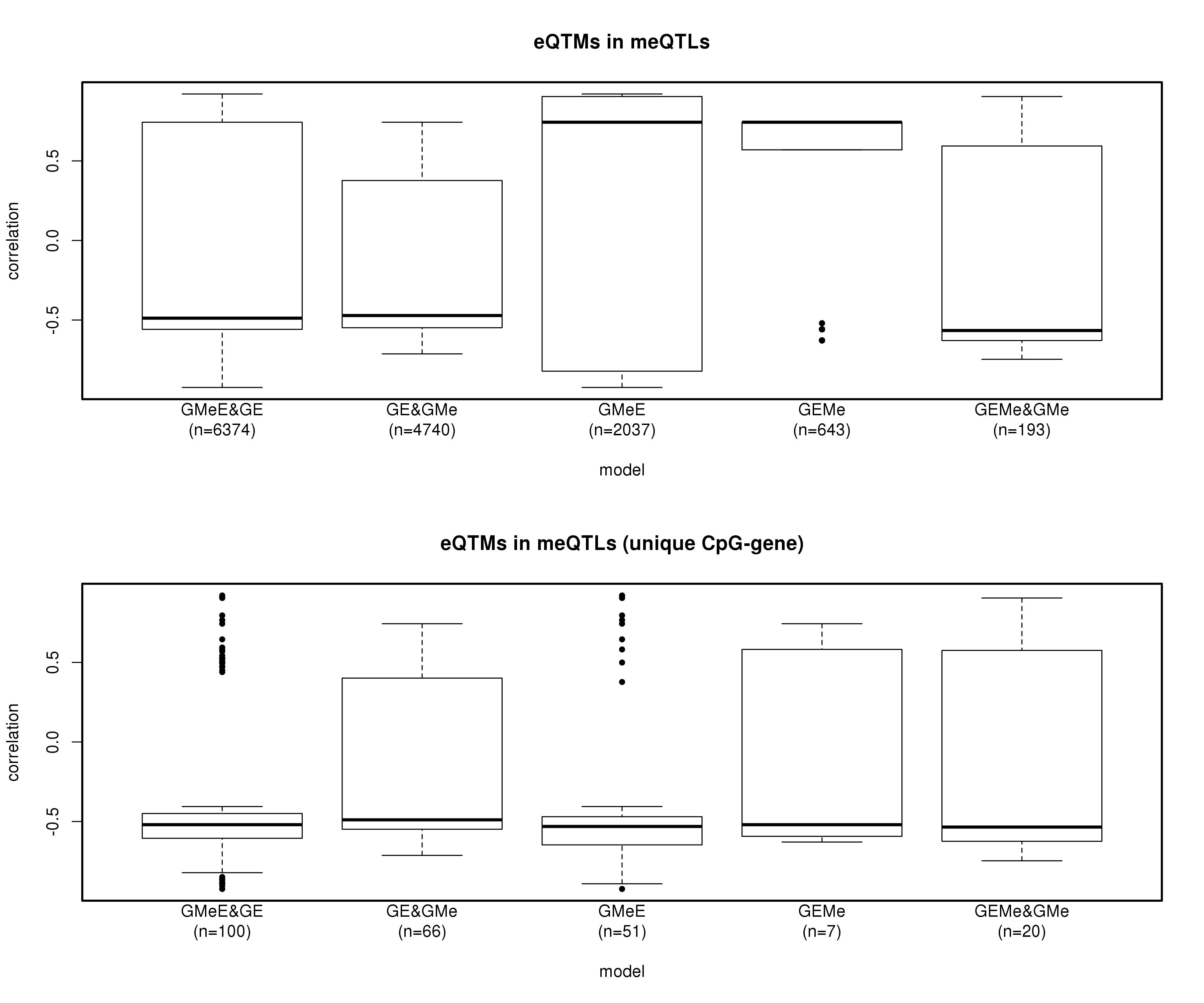

Supplement: Supplementary file 12 — Additional file 12: Figure 5: Distribution of the correlation between CpGs and genes (eQTMs) in meQTLs (top) and unique eQTMs in meQTLs (bottom) for the Normal group along the different models. [file 13148_2021_1148_MOESM12_ESM.png]

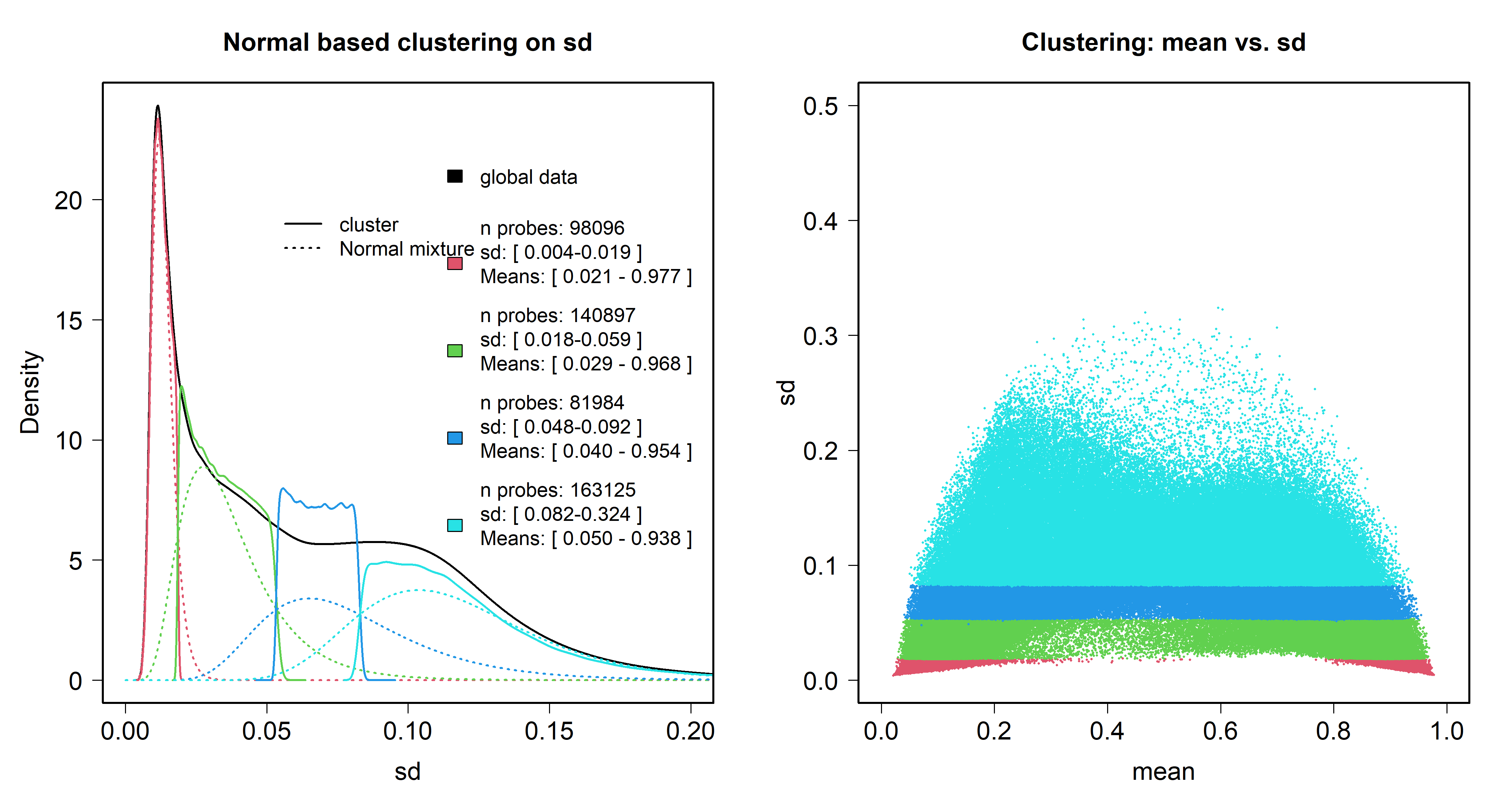

Supplement: Supplementary file 14 — Additional file 14: Figure 6. A) Mixture of normal distributions and clusters of CpGs according to the standard deviation (sd) of the beta-values. B) Distribution of CpGs according to the mean beta-value and standard deviation, with clusters colored. CpGs with sd < 0.05 were excluded from analysis. [file 13148_2021_1148_MOESM14_ESM.png]
